# Social stress is unlikely to play a major role in reproductive suppression of female subordinate naked mole-rats and Damaraland mole-rats

**DOI:** 10.1098/rsbl.2022.0292

**Published:** 2022-10-26

**Authors:** Daniel W. Hart, Nigel C. Bennett, Cornelia Voigt

**Affiliations:** Department of Zoology and Entomology, University of Pretoria, Private Bag X20, Hatfield 0028, South Africa

**Keywords:** mole-rats, stress, glucocorticoids, reproductive suppression

Medger [1] reviewed the mechanisms of reproductive suppression in two eusocial mole-rat species, the naked (*Heterocephalus glaber)* and Damaraland mole-rat (*Fukomys damarensis*) with a focus on the interplay of stress, glucocorticoids and reproductive activity in subordinate females. By referring to findings from other group-living mammals such as meerkats (*Suricata suricatta*) and olive baboons (*Papio anubi*), Medger [1] suggested that in these mole-rat species female subordinates (non-breeding colony members) are exposed to aggressive behaviours from dominant individuals, namely the queen (sole breeding female), causing elevated glucocorticoid levels, which lead to a block of reproduction. Medger [1] emphasizes the importance of environmental factors and colony stability in regulating stress in both species and extrapolates this to other social mole-rat species.

Medger [1] suggested that female subordinates of both species are exposed to substantial aggression by female breeders to prevent reproduction, leading to increased glucocorticoid levels. However, this conclusion is not supported by the literature, as to date, no study on unmanipulated and stable colonies of Damaraland mole-rats has observed apparent aggression between dominant breeding individuals and subordinates. Furthermore, previous observations on a small number of unmanipulated colonies of naked mole-rats showed that shoving (a proxy for aggression) by the queens was not more often directed toward high-ranking subordinate females, which are those that were to succeed them, than toward other colony members [2,3].

The studies cited by Medger [1] are all experimental in nature, as either the queen was removed from the colony, or unrelated males were introduced [4,5]. It is conceivable that such interference causes colony instability, which results in aggressive behaviours among colony members and possibly increased glucocorticoids. As pointed out by Medger [1], glucocorticoid levels do not differ between breeding and reproductively suppressed naked and Damaraland mole-rats [6,7]. The seasonal variations in glucocorticoids found in Damaraland mole-rats, particularly just after the rains [8], are likely the consequence of mobilization of carbohydrate reserves for the period of intense burrowing, as reported by Vullioud *et al*. [9], rather than variation in reproductive suppression.

Medger [1] raises an essential point that the main mechanisms of reproductive suppression in subordinate females differ between the two eusocial mole-rat species. The difference in the suppressive mechanisms likely results from the two species possessing different ovulatory modes; however, this was not explored [10,11]. Naked mole-rats are spontaneous ovulators and reproductive suppression, found in the form of anovulation, results from suppression of the onset of ovarian cyclicity in subordinate females, which therefore remain in a prepubertal stage [12]. By contrast, Damaraland mole-rats are induced ovulators, and anovulation in subordinate females occurs through a lack of copulation [11]. Consequently, such females remain in a prepubertal stage until virgin mating occurs, activating ovarian steroidogenesis, thereby priming the hypothalamic–pituitary–gonadal (HPG) axis and, consequently, inducing the onset of puberty [13]. These physiological differences explain why inbreeding avoidance, a major component of reproductive suppression [14], is effective in maintaining reproductive skew in Damaraland mole-rats, but not in naked mole-rats.

Medger [1] suggests that studies investigating the neuroendocrine mechanisms underlying reproduction can help understand the mechanism of reproductive suppression. However, Medger [1] fails to explore the differences between the two species. In spontaneously ovulating mammals, such as naked mole-rats, the positive feedback action of ovarian oestradiol leads to the release of gonadotropin-releasing hormone (GnRH), which stimulates the pre-ovulatory luteinizing hormone (LH) surge from the anterior pituitary. The neuropeptides, kisspeptin and RFamide-related peptide -3 (RFRP-3) are important regulators of gonadotropin release [15]. Zhou *et al*. [16] found that subordinate female naked mole-rats, which are anovulatory, possess significantly fewer kisspeptin-immunoreactive cells in the anterior hypothalamus compared to queens, suggesting that this neuron population is involved in the pre-ovulatory LH surge in naked mole-rats. Moreover, it implies that triggering the activation of these kisspeptin neurons is essential for the onset of reproductive activity. Peragine *et al*. [17] reported that RFRP-3 is crucial in suppressing puberty onset in naked mole-rats. Subordinate females show increased RFRP-3 expression in the dorsomedial hypothalamus compared to queens [17]. Further, treatment with exogenous RFRP-3 prevented puberty onset in subordinate females removed from their natal colony and allowed to enter puberty. Faykoo-Martinez *et al*. [18] identified several candidate genes, including those of the kisspeptin signalling pathway, which show a differential expression pattern in relation to reproductive status.

In induced ovulators, such as the Damaraland mole-rat, the mating stimulus activates kisspeptin neurons in the anterior hypothalamus, which leads to activation of GnRH neurons and, consequently, to ovulation [15,19]. Several recent studies in female Damaraland mole-rats have identified differential hypothalamic gene expression patterns of neuropeptides involved in activating GnRH neurons according to the females' reproductive status. Queens have significantly more *Kiss1*-expressing cells, increased neurokinin B and decreased dynorphin gene expression in the arcuate nucleus of the hypothalamus compared to subordinate females [20–22]. Like naked mole-rat queens, Damaraland mole-rat queens have significantly fewer RFRP-3-expressing cells within the hypothalamus than subordinate females [20].

Medger [1] also eluded to the variation in the longevity between breeders and subordinates in social mole-rat species, which is a point of interest for many researchers [23–28]. Medger [1] suggested that the lower oxidative damage found in breeding females of the Damaraland mole-rat contributes to their longer lifespan [29] but does not mention that the opposite trend is seen in naked mole-rats [28]. Further, Medger [1] argues that high levels of glucocorticoids may contribute to the lower life expectancy of non-breeders. In the absence of empirical data, this remains pure speculation.

In conclusion, the existing body of research on naked and Damaraland mole-rats does not support the view that stress and an associated increase in glucocorticoids due to intra-sexual aggression plays a major role in reproductive suppression. In Damaraland mole-rats inbreeding avoidance plays a significant role in reproductive suppression, whereas in naked mole-rat elevated prolactin in subordinates of both sexes may bring about social suppression [14,30]. However, the importance of induced stress may play a significant role in reproductive suppression and colony stability in other social and eusocial mole-rat species [31–33].

## Data Availability

This article has no additional data.
